# Calculating the Electrical Conductivity of Graphene Nanoplatelet Polymer Composites by a Monte Carlo Method

**DOI:** 10.3390/nano10061129

**Published:** 2020-06-08

**Authors:** Chao Fang, Juanjuan Zhang, Xiqu Chen, George J. Weng

**Affiliations:** 1 Department of Electrical and Electronic Engineering, Wuhan Polytechnic University, Wuhan 430023, China; yyohjh@sina.com (C.F.); cxqdhl@sina.cn (X.C.); 2 Key Laboratory of Mechanics on Environment and Disaster in Western China, The Ministry of Education of China, Lanzhou University, Lanzhou 730000, China; zhangjuanjuan@lzu.edu.cn; 3 Department of Mechanics and Engineering Science, College of Civil Engineering and Mechanics, Lanzhou University, Lanzhou 730000, China; 4 Department of Mechanical and Aerospace Engineering, Rutgers University, New Brunswick, NJ 08903, USA

**Keywords:** electrical conductivity, graphene polymer nanocomposites, Monte Carlo simulations

## Abstract

Electrical conductivity is one of several outstanding features of graphene–polymer nanocomposites, but calculations of this property require the intricate features of the underlying conduction processes to be accounted for. To this end, a novel Monte Carlo method was developed. We first established a randomly distributed graphene nanoplatelet (GNP) network. Then, based on the tunneling effect, the contact conductance between the GNPs was calculated. Coated surfaces (CSs) were next set up to calculate the current flow from the GNPs to the polymer. Using the equipotential approximation, the potentials of the GNPs and CSs met Kirchhoff’s current law, and, based on Laplace equation, the potential of the CSs was obtained from the potential of the GNP by the walk-on-spheres (WoS) method. As such, the potentials of all GNPs were obtained, and the electrical conductivity of the GNP polymer composites was calculated. The barrier heights, polymer conductivity, diameter and thickness of the GNP determining the electrical conductivity of composites were studied in this model. The calculated conductivity and percolation threshold were shown to agree with experimental data.

## 1. Introduction

Graphene, due to its one-atom-thick 2D structure and excellent performance, has attracted extensive interest in recent years [1,2,3]. Composite materials based on graphene have excellent thermal and electrical conductivity [4,5], thermal stability [6], mechanical properties [7,8], optical characteristics [9,10], and polymer flexibility [11]. Therefore, graphene-based composites are becoming important parts of the field of graphene application. They have shown excellent performance in energy storage [12], liquid crystal devices [13], electromagnetic interference shielding [14], biomaterials [15], sensing materials [16], and electrocatalysts [17], among others.

The conductivity of a polymer can be greatly improved by using graphene as the conductive filler. Most polymers have a low conductivity of about 10^−10^ S/m, while the conductivity of graphene can be as high as 10^4^–10^5^ S/m [18]. It is known that graphene-based nanocomposites have a low percolation threshold and exhibit a preferable conductive property. For instance, in a very recent experiment, Nistal et al. [19] reported a high electrical conductivity (~40 S/m) with a percolation limit of 1.18 vol.%. When the filler reached a certain concentration, the nanocomposite changed from insulators to conductors, and the electrical conductivity rose sharply by a range of 10 orders of magnitude. This well-known percolation phenomenon is commonly found in particle-filled polymer composites. The percolation threshold is closely related to the shape, distribution, orientation, and other factors of fillers that determine the formation of percolation path through the system. Therefore, the percolation threshold has become a main performance metric of graphene nanoplatelet (GNP) polymer composites.

The issues of percolation are complicated, and they can usually be studied by two methods: One is continuum theory based on micromechanics and the other is the Monte Carlo (MC) method. Based on the continuum model, Bruggeman’s effective-medium theory [20], as formulated by Maxwell’s far-field matching by setting the property of the reference medium to that of the effective medium (i.e., the composite) in [21], can provide the percolation threshold and an effective conductivity. Based on this approach, Xia et al. [22,23] studied the electrical conductivity of graphene composite foams and the influence of the orientation of graphene fillers on the conductivity of highly aligned graphene composites. A two-scale composite model made of graphene-rich and graphene-poor regions was reported to study the electrical conductivity of the agglomerated composite [24]. The DC formulation has also been extended to the AC condition by replacing DC conductivity with frequency-dependent complex conductivity [25,26]. In a separate development based on the excluded-volume theory, the percolation thresholds of composite with fillers including cylinders, spheroids, and spherocylinders can be described as a function of the excluded-volume of the convex cores [27]. The percolation behavior of graphene flakes has also been interpreted using a contact–volume argument [28].

The MC method is a statistical experimental method that is more suitable for solving random filler distribution problems than the continuum approach. However, the MC method often requires a large amount of calculations and is time-consuming, because it is difficult to calculate the space distance, especially for the irregular shape of fillers. Among the very limited number of studies, Oskouyi and Mertiny [29] developed a 3D continuous Monte Carlo model of a composite with the filler of 2D circular nano-disks. Wang and Jayatissa [30] carried out a 3D simulation of nanocomposite filled with rectangular platelets of hard-core soft-shells. Considering the tunneling effect, Zabihi and Araghi [31] investigated the effect of the orientation of graphene on the conductivity of nanocomposites.

In general, it is more difficult to establish an MC model for GNP nanocomposites than for carbon nanotube (CNT) nanocomposites. The MC model for CNT composites only requires the calculation of the distance between two CNTs of random distribution, which can be replaced by the distance between two separate line segments in space. For GNP nanocomposites, if the GNP is regarded as a short cylinder or a rectangular platelet with a small thickness, the calculation of distance is very complex. For this reason, a GNP is usually treated as a two-dimensional circular disk, and the distance between GNPs is replaced by the distance between round faces, which can be calculated by a numerical iterative algorithm [29,31]. Even with this simplification, the distance calculation in GNP nanocomposites is still extremely time-consuming, because there are usually at least thousands of GNPs in the representative volume element (RVE, usually taken to be of cuboidal shape).

In this work, we established a 3D Monte Carlo model to calculate the conductivity of GNP composite materials. The computation was optimized by regarding GNPs as a set of points whose distance from a round face in space was less than or equal to *ρ* (a given positive number; see Equation (1)), so a GNP is a three-dimensional filler with a flexible adjusted thickness of 2*ρ,* the distance between which could be reduced to the distance between two round faces minus 2*ρ*. By comparing the contact conductance, graphene conductance, and the interlayer conductance, we found that GNPs were approximately equipotential if the thickness of the GNP was much smaller than its diameter. With the equipotential approximation and the walk-on-spheres (WoS) method, the conductivity of the GNP composite could be obtained near the percolation threshold.

## 2. Monte Carlo Method for Graphene Nanoplatelet Polymer Composites

### 2.1. The GNP Networks

A GNP consists of a short stack of graphene sheets, so it was approximated by a short, cylinder element here. We built a mathematical model to describe the shape of a GNP, as shown in Equation (1):

(1) |f−g|≤ρ

where *g* and *ρ* are point sets in three-dimensional space and a given positive number, respectively. If *g* is a set of isolated points, then *f* that satisfies Equation (1) is a set of spheres with radius *ρ*; if *g* is a set of line segments with length *L*, then *f* comprises CNTs with radius *ρ* and length *L* + 2*ρ* [32]; similarly, if *g* is a set of round faces with diameter of *D*’, then *f* comprises a GNP with diameter of *D* = *D*’ + 2*ρ* and a thickness of 2*ρ*.

**Figure 1:**
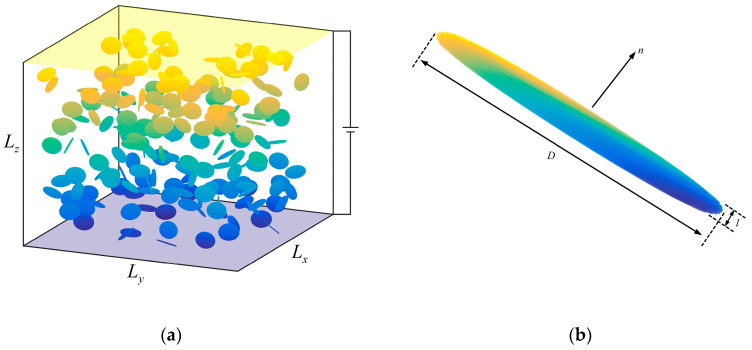
Schematics of (**a**) graphene nanoplatelet (GNP) network in a representative volume element (RVE) and (**b**) position and orientation of the GNP.

In Figure 1a, GNP fillers were randomly generated in RVE, the size of which was *L_x_* × *L_y_* × *L_z_*. Figure 1b shows the shape of a GNP filler *f_i_*, and the center coordinates of the corresponding round face *g_i_* is:

(2) $$\begin{array}{c} X_{i}=L_{x}\times rand,\\ Y_{i}=L_{y}\times rand,\\ Z_{i}=L_{z}\times rand. \end{array}$$

where *i* is the index of the *i*th GNP and ‘*rand*’ is a random number of uniform distributions on an interval of (0, 1) that is generated by a computer. The normal direction of round face *g_i_* is:

(3) $$\begin{array}{c} n_{i}=\left(\sin\theta_{i}\cos\varphi_{i},\sin\theta_{i}\sin\varphi_{i},\cos\theta_{i}\right),\\ \theta_{i}=\cos^{-1}\left(2\times rand-1\right),\\ \varphi_{i}=2\pi \times rand. \end{array}$$

where *θ_i_* and *φ_i_* are the azimuthal and the polar angles of the normal direction, respectively. In order to investigate the influence of thickness and diameter of the GNPs on the conductivity of composite, it was assumed that GNPs in the RVE had the same size. *z* = *L_z_* plane and *z* = 0 plane represent the positive and negative electrode, respectively. The distribution of the GNP was consistent with the periodic arrangement of *L_x_* in the *x* direction and *L_y_* in the *y* direction.

### 2.2. Graphene Conductance, Contact Conductance and Interlayer Conductance

**Figure 2:**
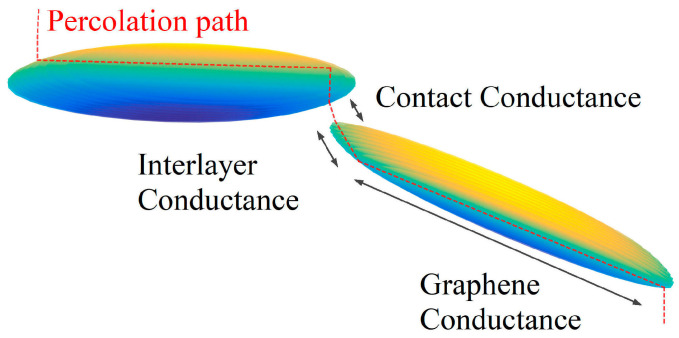
A diagram of *G_graphene_* (the conductance of graphene), *G_interlayer_* (the conductance between the top and bottom layer of a GNP), and *G_contact_* (the tunneling conductance between a pair of GNPs).

Figure 2 shows a part of a percolation path. It can be seen that the conductance of the percolation path is composed of three parts in series: *G_graphene_*, *G_contact_*, and *G_interlayer_*; *G_graphene_* is the conductance of graphene and can be expressed by:

(4) $$G_{graphene}=\frac{2e^{2}N_{chan}}{h},$$

where 2*e*^2^*/h* denotes the quantized conductance and *N_chan_* is the number of channels for graphene that is proportional to the size of graphene *N_chan_ = aD*, where a is constant and we took *a* = 0.01 nm^−1^ [33]. For *D* = 1 μm, one can get a sheet resistance 1/*G_graphene_ =* 1295 Ω/□, which is reasonable [34,35]. Here, electronic scattering behavior was ignored because *D* in our calculation was far less than the mean free path, which could exceed 28 μm [2].

The tunneling effects between GNPs were considered here. Once the distance between GNPs is within a few nanometers, tunneling can occur. Because the tunneling current exponentially increases with the decreasing distance, electron tunneling mainly occurs between the nearest two graphenes, which come from two different GNPs. Venugopal et al. [36] pointed out that only one or two layers of a graphene stack take the role of conducting current. The GNPs only interact with the first few layers, so we could calculate the contact conductance between the nearest two graphenes instead of that between GNPs. The tunneling effect could only be ignored if the distance between two GNPs exceeds the cut-off distance of 1.4 nm [37,38]. The contact conductance is the tunneling conductance between a pair of GNPs, and can be expressed as:

(5) $$G_{contact}=G_{graphene}exp\left[-\frac{\sqrt{8m_{e}\Delta E}max\left(d_{GNPs},d_{vdW}\right)}{\hbar }\right],$$

where Δ*E* is the barrier height and *d_GNPs_* is the distance between two GNPs. According to definition of the GNP in Equation (1), *d_GNPs_* is equal to the distance between the two round faces minus the thickness of the GNP. The van der Waals separation distance *d_vdW_* is the minimum distance between GNPs [39].

Thus the contact conductance between GNPs is tunneling conductance. The types of contacts between GNPs are point-to-point and point-to-face contact, so the computation of contact conductance based on quantum conductance is similar to the tunneling conductance between CNTs because of the small contact area. *G_interlayer_* is the conductance between the top and bottom layer of a GNP, and the type of contacts between the graphene layers is face-to-face contact. Because of the large contact area, *G_interlayer_* is much larger than *G_contact_* and cannot be calculated by Equation (5). Here, we analyzed *G_interlayer_* by the law of resistance*. G_interlayer_* varies inversely with thickness [40] and can be expressed as:

(6) $$G_{interlayer}=\frac{\sigma_{3}\pi D^{2}}{4l},$$

where *σ*_3_ is the conductivity in the vertical direction of the GNP.

According to Equation (5), it can be estimated that *G_contact_* is rather smaller than the conductance of the monolayer graphene *G_graphene_* on account of *d_vdW_* (between 0.3 and 0.4 nm [37,41,42]) and Δ*E* (several eV [43]). According to Equation (6), *G_interlayer_*/*G_graphene_ = σ*_3_*πD*^2^/4*σ*_1_*ld_vdW_*. Because *σ*_3_ = 0.001*σ*_1_ [22] or 0.1*σ*_1_ [18], *D* > *l*, and *D* is generally on the micron level, which is about four orders of magnitude larger than *d_vdW_*. We think that in general, *G_graphene_ < G_interlayer_*. In this way, the relationship between the three conductivities can be obtained as follows: *G_contact_* ≪ *G_graphene_* < *G_interlayer_*. Thus, each GNP is approximately equipotential.

### 2.3. Potential Equations of GNPs

**Figure 3:**
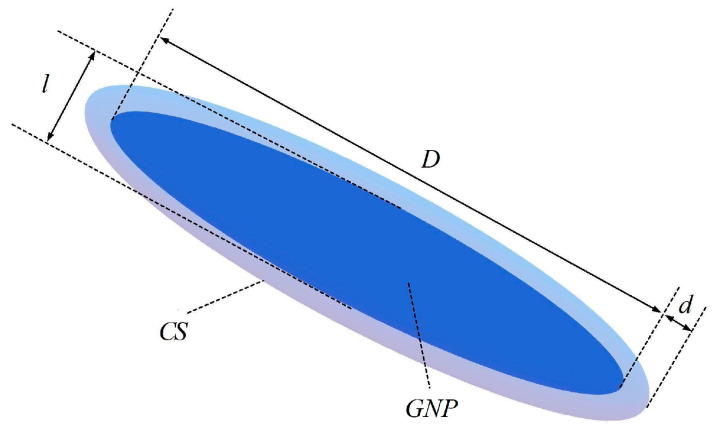
A coated surface (CS) on a GNP.

Based on equipotential approximation, the potential equations of GNP can be constructed. For simplicity, we used *U*^1^, *U*^2^, and *U*^3^ to represent the potential of the GNP, the coated surface (CS), and the electrode, respectively. *U*^1^ and *U*^2^ are both an *N* × 1 matrix, and *U*^3^ is a 2 × 1 matrix. In order to compute the current flowing into the polymer, the CS is introduced, as shown in Figure 3. The CS is considered as the surface of *f* defined in Equation (1), taking *ρ* = *l*/2 + *d*, where *d* is the distance between the GNP and the corresponding CS. Next, we built the equations for *U*^1^ and *U*^2^.

For the *i*th GNP, based on the law of Kirchhoff, we have:

(7) $$\overset{N}{\underset{j=1}{\sum }}G_{ij}^{11}\left(U_{i}^{1}-U_{j}^{1}\right)+\overset{N}{\underset{j=1}{\sum }}G_{ij}^{12}\left(U_{i}^{1}-U_{j}^{2}\right)+\overset{2}{\underset{j=1}{\sum }}G_{ij}^{13}\left(U_{i}^{1}-U_{j}^{3}\right)=0,i=1,2,\ldots ,N$$

The sum of these three currents is zero: the tunneling current between GNPs, the direct current from the GNPs to the CS, and the tunneling current from the GNPs to the electrodes. In this expression, *G*^11^ is an *N* × *N* matrix of contact conductance, the elements of which can be evaluated as:

(8) $$G_{ij}^{11}=\{\begin{array}{c} 0,i=j\\ G_{0}N_{chan}exp\left[-\frac{\sqrt{8m_{e}\Delta E}max\left(d_{ij}^{11},d_{vdW}\right)}{\hbar }\right],i\neq j \end{array},$$

where $d_{ij}^{11}$ is the distance between GNPs, with:

(9) $$d_{ij}^{11}=d_{ij}^{g}-l,$$

where $d_{ij}^{g}$ is the distance between the round face *g_i_* and *g_j_*. When the thickness *l* changes, one does not need to calculate $d_{ij}^{g}$ repeatedly to get $d_{ij}^{11}$. *G*^12^ is also an *N* × *N* matrix, the elements of which are $G_{ij}^{12}$, the direct conductance between the *i*th GNP and the *j*th CS:

(10) $$G_{ij}^{12}=\{\begin{array}{c} \sigma_{0}S/d,i=j\\ 0,i\neq j \end{array},$$

where *S* is the surface area of the GNP, *S* = π*D*^2^/2 + π^2^*Dl*/2 + π*l*^2^, and *σ*_0_ denotes the conductivity of the polymer. Trying to avoid the case in which the CS intersects with the GNPs, *d* needs to be small enough, so we took *d* = *d_vdW_*/2 here. An *N* × 2 matrix *G*^13^ denotes the contact conductance between GNPs and electrodes, in consideration of van der Waals interactions, and $G_{ij}^{13}$ can be written as:

(11) $$G_{ij}^{13}=G_{0}N_{chan}exp\left[-\frac{\sqrt{8m_{e}\Delta E}max\left(d_{ij}^{13},d_{vdW}\right)}{\hbar }\right],$$

where $d_{ij}^{13}$ denotes the distance between the GNPs and the electrodes.

In this way, Equation (7) can be evolved in linear equations as:

(12) $$U^{1}=Q^{11}U^{1}+Q^{12}U^{2}+Q^{13}U^{3},$$

where the elements of the matrix *Q*^11^, *Q*^12^, and *Q*^13^ are:

(13) $$Q_{ij}^{11}=\frac{G_{ij}^{11}}{\sum_{j=1}^{N}\left(G_{ij}^{11}+G_{ij}^{12}\right)+\sum_{j=1}^{2}G_{ij}^{13}},\;Q_{ij}^{12}=\frac{G_{ij}^{12}}{\sum_{j=1}^{N}\left(G_{ij}^{11}+G_{ij}^{12}\right)+\sum_{j=1}^{2}G_{ij}^{13}},\;Q_{ij}^{13}=\frac{G_{ij}^{13}}{\sum_{j=1}^{N}\left(G_{ij}^{11}+G_{ij}^{12}\right)+\sum_{j=1}^{2}G_{ij}^{13}}$$

### 2.4. Potential Equations of CS

Assuming that the matrix is an isotropic medium with uniform conductivity and permittivity, the electrical potential in matrix is determined by the Poisson equation:

(14) $$\begin{array}{c} \nabla^{2}u\left(r\right)=-\frac{\rho }{\varepsilon },r\in \Gamma \\ u\left(r\right)=U\left(r\right),r\in \partial \Gamma \end{array}.$$

where *Γ* and ∂*Γ* denote the matrix domain and its boundary, respectively. *ρ* is the volume charge density, and *ε* is the dielectric constant of polymer. Carriers injected from the electrodes are captured by the traps formed by the defects, interfaces, and impurities in the polymer medium, all of which turn out to be the volume charge. Our model calculates the conductivity of the composite under a constant electric field, so the volume charge in shallow traps migrates to the surface of the material under steady voltage, while the volume charge in deep traps is immovable and mainly concentrated near the electrode. On the one hand, the volume charge of non-uniform distribution makes it difficult to solve the Poisson equation; on the other hand, the space charge may vary the electric field distribution and carrier mobility, thus leading to a change in conductivity of the polymer. Therefore, in order to simplify the calculation, Equation (14) can be simplified to Laplace equation, and the effect of the volume charge on the calculation results could be reflected by setting a different conductivity of the polymer:

(15) $$\begin{array}{c} \nabla^{2}u\left(r\right)=0,r\in \Gamma \\ u\left(r\right)=U\left(r\right),r\in \partial \Gamma \end{array}.$$

with:

(16) $$U\left(r\right)=\{\begin{array}{c} U_{i}^{1},r\in GNP_{i},i=1,2,\ldots ,N,\\ U_{j}^{3},r\in E_{j},j=1,2. \end{array}$$

where *GNP_i_* is *f_i_* (defined by Equation (1)) and *E*_1_ and *E*_2_ denote two electrodes. In particular, if *r* is in the overlapping domain, then *U*(*r*) is the average potential of coincident GNPs.

**Figure 4:**
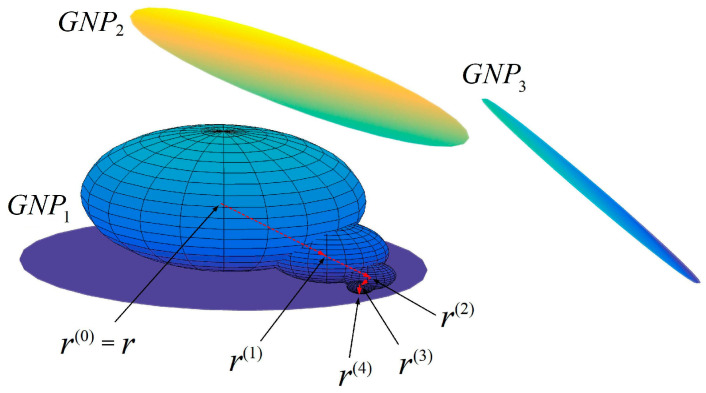
A random walk process on a sphere.

The solutions to Equation (15) can be obtained by WoS Monte Carlo simulations. Figure 4 shows an example, where *r* is the point where the potential is required in matrix. First, one draws a ball with *r* as its center. The ball needs to be big enough but cannot intersect any boundary ∂*Γ*. Next, a random point *r*^(1)^ is generated on the surface of the ball. Then this step is repeated, and a trace is indicated with the red arrow. When the obtained *r* is close enough to a GNP or electrode, this random walk is stopped.

According to law of large numbers:

(17) $$u\left(r\right)=\underset{M\to \infty }{lim}\frac{1}{M}\overset{M}{\underset{k=1}{\sum }}U\left(\partial \Gamma_{k}\right),$$

where $\partial \Gamma_{k}$ is the boundary that is closest to the terminal point and *k* is the ordinal number of random walk [44,45]. The potential at *r* is the expected value of the potential at the end of random walks. Equation (17) can be simplified to:

(18) $$u\left(r\right)=\underset{M\to \infty }{lim}\frac{1}{M}\overset{M}{\underset{k=1}{\sum }}\left[\overset{N}{\underset{j=1}{\sum }}U_{j}^{1}P\left\{GNP_{j}|r\right\}+\overset{2}{\underset{j=1}{\sum }}U_{j}^{3}P\left\{E_{j}|r\right\}\right],$$

where *P*{*GNP_j_*|*r*} and *P*{*E_j_*|*r*} are Boolean variables that denote the events reaching *GNP_j_* and *E_j_,* respectively.

When putting *r* on a CS, Equation (18) can be expressed by:

(19) $$U^{2}=Q^{21}U^{1}+Q^{23}U^{3},$$

where the elements of the matrix *Q*^21^ and *Q*^23^ are:

(20) $$Q_{ij}^{21}=\underset{M\to \infty }{lim}\frac{1}{M}\overset{M}{\underset{k=1}{\sum }}P\left\{GNP_{j}|CS_{i}\right\},\;Q_{ij}^{23}=\underset{M\to \infty }{lim}\frac{1}{M}\overset{M}{\underset{k=1}{\sum }}P\left\{E_{j}|CS_{i}\right\}.$$

For the detailed process of the WoS method to calculate *Q*^21^ and *Q*^23^, refer to [32].

### 2.5. The Conductance of the Nanocomposite

Equations (12) and (19) are combined to get:

(21) U=QU

where:

(22) $$U=\left[\begin{array}{c} U^{1}\\ U^{2}\\ U^{3} \end{array}\right],Q=\left[\begin{array}{ccc} Q^{11} & Q^{12} & Q^{13}\\ Q^{21} & Q^{22} & Q^{23}\\ Q^{31} & Q^{32} & Q^{33} \end{array}\right].$$

where *Q*^22^ is the *N* × *N* zero matrix, *Q*^31^ and *Q*^32^ are both 2 × *N* zero matrixes, and *Q*^33^ is a 2 × 2 unit matrix. The linear Equation (21) can be solved through an iterative approach.

We constructed a plane, *z* = *d* = *d_vdW_*/2, that is parallel to the electrode, and the current of the composite material is equal to the current flowing through the plane, which can be calculated as:

(23) $$I=\frac{L_{x}L_{y}\sigma_{0}\left(U_{z=d}-U_{1}^{3}\right)}{d}+\overset{N}{\underset{i=1}{\sum }}G_{i1}^{13}\left(U_{i}^{1}-U_{1}^{3}\right),$$

where *U_z_*_= *d*_ can be computed by the WoS method. The total current is the sum of the direct current from the plane to electrode 1 and the tunneling current from the GNPs to electrode 1. Moreover, the conductivity of the composite can be given as:

(24) $$\sigma =\frac{IL_{z}}{\left(U_{2}^{3}-U_{1}^{3}\right)L_{x}L_{y}}.$$

## 3. Results and Discussion

**Figure 5:**
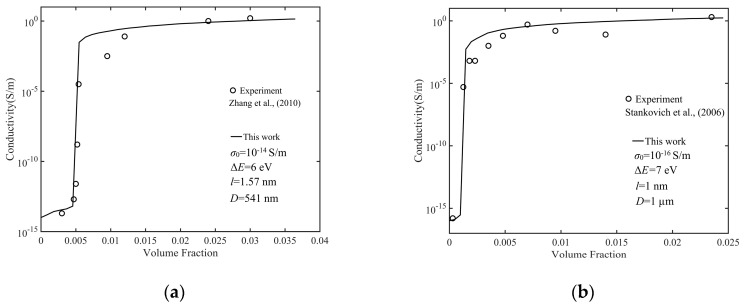

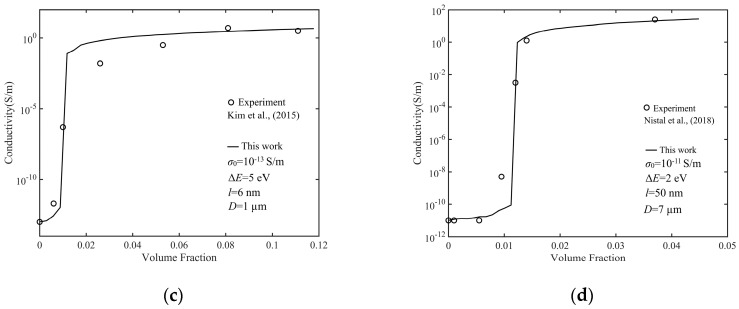
Numerical simulation results and experimental data of conductivity: (**a**) with Zhang et al. [46], (**b**) with Stankovich et al. [18], (**c**) with Kim et al. [47], and (**d**) with Nistal et al. [19].

Only four adjustable parameters are used in the whole calculation process: polymer conductivity *σ*_0_, barrier height Δ*E*, thickness *l*, and the diameter *D* of the GNP. Figure 5 shows the volume fraction dependence of the conductivity of the GNP composite with various parameters. It can be seen that four sets of theoretical results fit well with the experimental data [18,19,46,47]. When the volume fraction was zero, the conductivity of the GNP composite was equal to that of the polymer; when the volume fraction increased, the conductivity increased slowly, because the filler was approximately equipotential and its role was to shorten the distance between two electrodes filled with the polymer. When no percolation path was formed in the material, there were two cases: (1) There were GNPs close to electrode 1 (less than cut off distance), where the potential of the GNPs was basically the same as that of electrode 1 because these GNPs did not form a percolation path; (2) there was no GNP close to electrode 1, so $G_{i1}^{13}$ was very small or even less than the conductivity of the polymer. In both cases, according to Equation (23), the tunneling current was almost zero, so the total current and the conductivity of the GNP composite were determined by direct current. When no percolation path was formed, with the GNP concentration increasing, the increase of *U_z_*_= *d*_ resulted in the increase of the total current, which caused the increase of conductivity by about one order of magnitude. This phenomenon was similar to that of the CNT composite, and the difference was that the conductivity of the CNT composite increased by about two or three orders of magnitude at this stage [32]. It can be seen that due to the differences in the shapes of the GNP and the CNT, the contribution of the GNP to conductivity was weaker than that of the CNT before the formation of the percolation path. When the concentration of the GNP was above the percolation threshold, a conductive network was formed between the two electrodes, and the potential of the GNP, which was close to electrode 1 (less than cut off distance), suddenly increased, resulting in a large tunneling current. Because the contact conductance between GNPs on a percolation path is greater than the direct conductance between GNPs, the tunneling current was much greater than the direct current, which resulted in a sharp rise in the conductivity. The tunneling current was related to the conductive network density and the number of GNPs on one path. With the further addition of GNPs, the number of percolation paths increased, and there may have been a shorter percolation path that brought about a further increase of the tunneling current and the conductivity. Similarly, above the percolation threshold, the increment of the conductivity of the GNP composite was smaller than that of the CNT composite [32], which might be attributable to the geometric properties of GNPs and CNTs.

**Figure 6:**
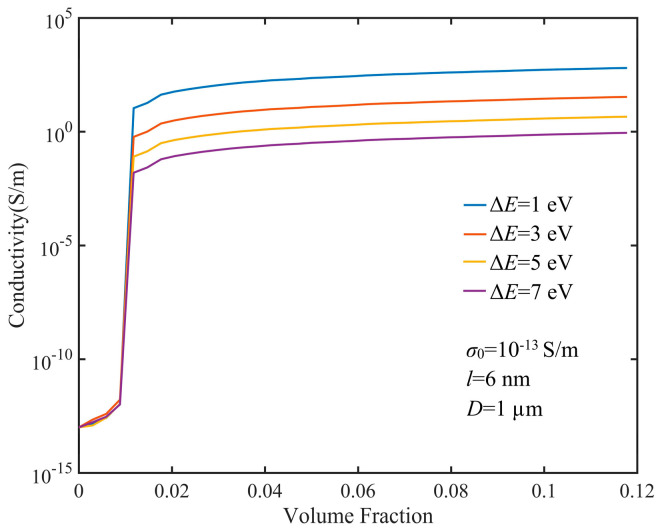
Tunneling effects of different barrier heights on the conductivity of GNP composites.

Due to the influence of barrier height on contact conductance, we calculated the full range of conductivity of the GNP composite with various barrier heights, as shown in Figure 6. We found that the barrier height affected the conductivity above the percolation threshold, but it made no difference to the conductivity below the percolation threshold. This was because there was a non-zero tunneling current only when the percolation path was formed in the composite, which was affected by the barrier height. There was a tunneling effect between a close pair of GNPs. According to Equation (5), tunneling conductance is related to the width and height of a barrier. Barrier height is the difference between the work function of a GNP and the medium between GNPs, which may be a polymer or an interphase formed by molecular interaction between the GNP and the polymer. The tunneling effect makes the contact conductance exponentially decrease with the increase of barrier height. It is clear that the conductivity of a composite is determined by contact conductance after the percolation threshold. In addition, barrier height does not change the percolation threshold of GNP composites, but for CNT composites, the percolation threshold slightly increases with increases of barrier height [32]. The reason for this phenomenon is that the gap between fillers in a CNT network has a certain randomness. As barrier height increases, a part of the contact conductance between CNTs with a larger distance decreases sharply, which causes a percolation path break. Therefore, with an increase of barrier height, the number of percolation paths decreases or even disappears, leading to a slight rise in the percolation threshold of CNT composites. In a GNP network, most of the neighboring GNPs forming percolation paths overlap with each other. In this way, almost all the effective distance between overlapped GNPs takes the minimum value *d_vdW_*. With the increase of barrier height, no percolation path fracture occurs. Therefore, barrier height does not affect the percolation threshold of a GNP composite.

**Figure 7:**
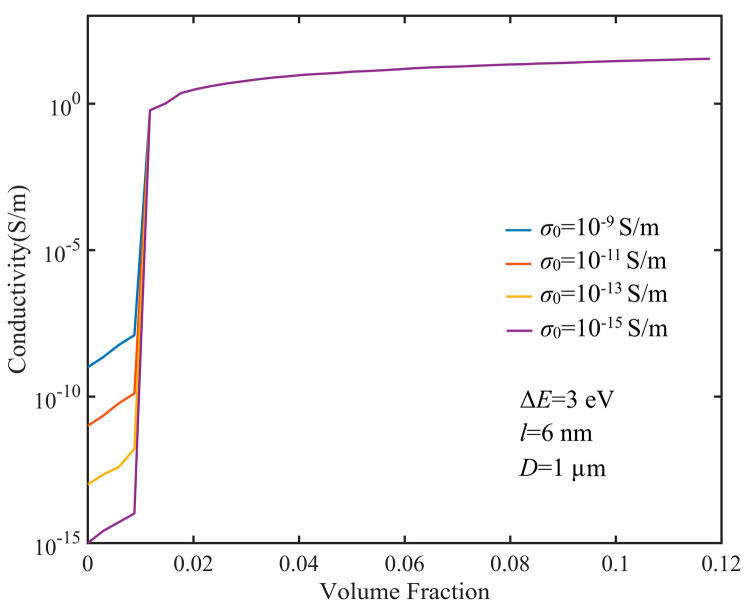
Electrical conductivity of GNP composites of different polymer conductivities.

The influence of polymer conductivity on the conductivity of GNP composites is illustrated in Figure 7. We took *σ*_0_ = 10^−15^–10^−9^ S/m. It could be seen that the matrix conductivity only changed the part of conductivity below the percolation threshold. For instance, *D* = 1000 nm, *l* = 10 nm, Δ*E =* 3 eV, $d_{i1}^{13}$ = *d_vdW_*, and *σ*_0_ = 10^−9^ S/m; on the basis of Equation (11), $G_{i1}^{13}$ = 1.74 × 10^−6^ S. The direct conductance was *L_x_L_y_σ*_0_/*d* = 5.8 × 10^−10^ S for *L_x_* = *L_y_* = 10 μm, which was much less than contact conductance. As a result, once the conductive network was established, the current of the GNP composites was determined by the tunneling current, and the conductivity was determined by contact conductance. The percolation threshold was closely associated with the formation of the conductive network in the composite without being influenced by the conductivity of the polymer.

**Figure 8:**
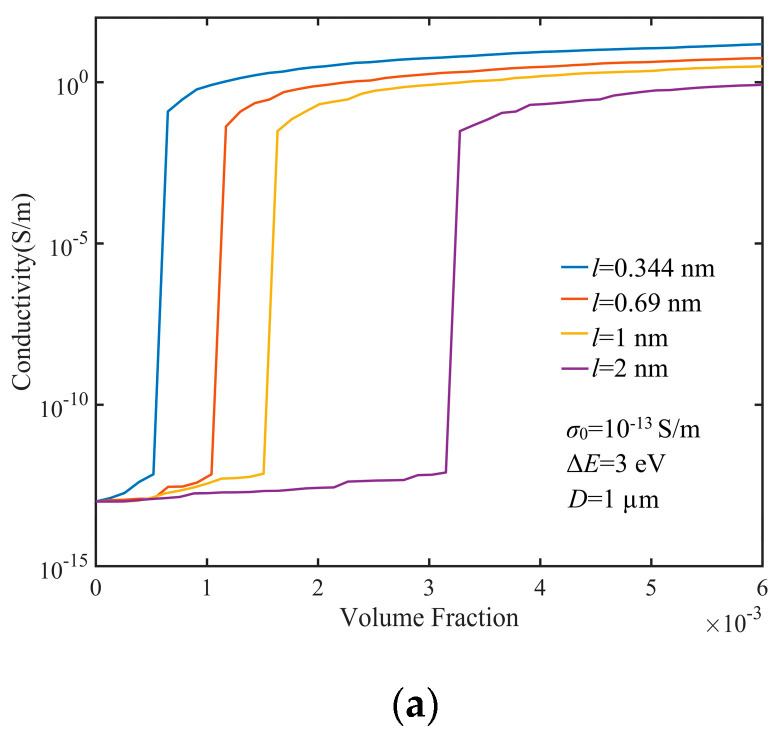

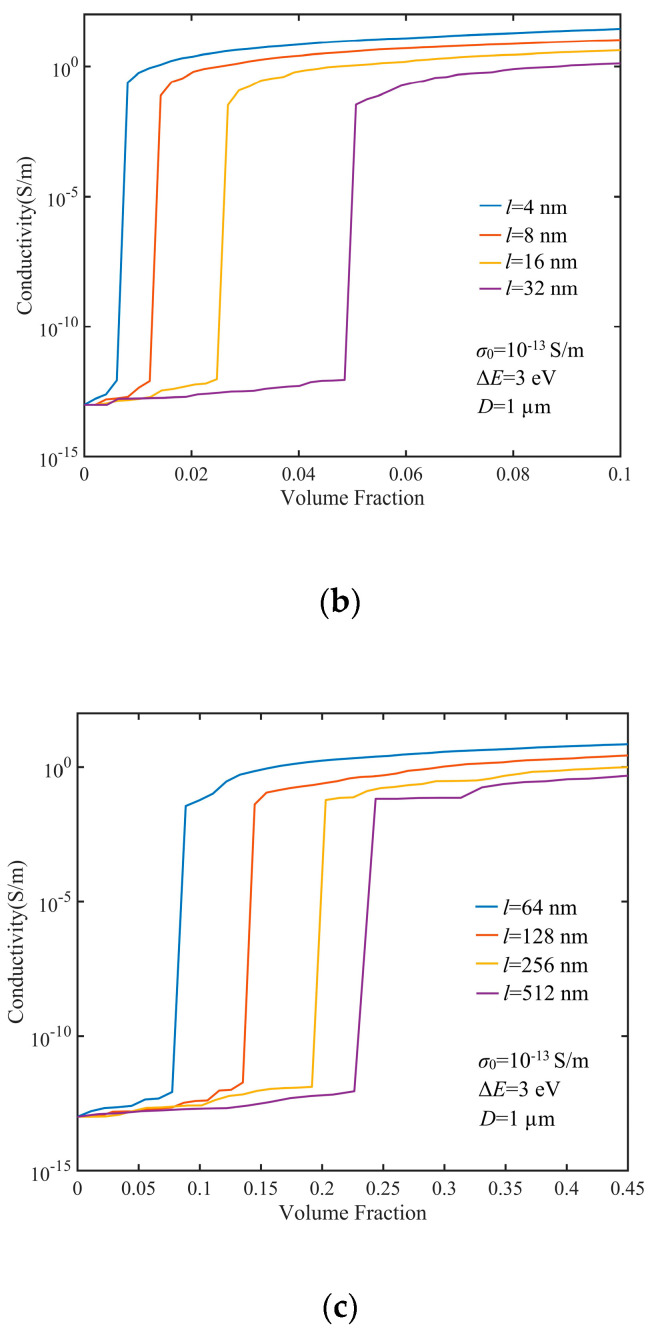
Electrical conductivity of GNP composites with different GNP thicknesses: (**a**) *l* = 0.344, 0.69, 1, and 2 nm; (**b**) *l* = 4, 8, 16, and 32 nm; and (**c**) *l* = 64, 128, 256, and 512 nm.

In order to analyze the impact of aspect ratio of the filler GNP on the percolation threshold, the conductivities of composites with different thicknesses of GNPs were calculated, as shown in Figure 8a–c. Thickness changed in the range of 0.344–256 nm. The results suggested that the percolation threshold increased as *l* increased. If *l* < *D*, the number of GNPs needed to form percolation paths did not change with *l,* so when *l* was very small, the lower concentration of GNPs could form percolation paths. With *l* increasing, the concentration required to form percolation paths increased correspondingly. As the volume fraction of GNPs increased, more percolation path formation made the conductivity of the composite increase. Because of the randomness of the Monte Carlo method, the conductivity curve of the composite with the GNP volume fraction was not smooth.

**Figure 9:**
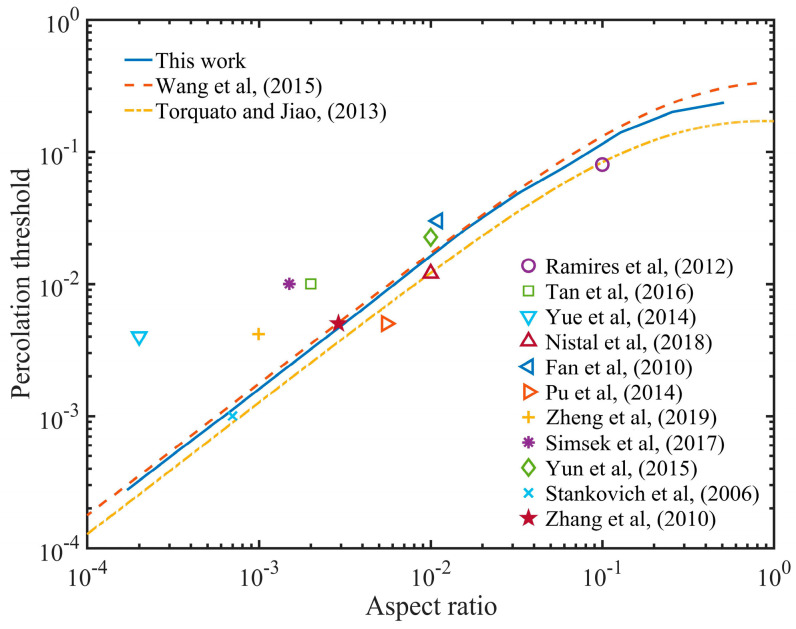
Percolation threshold versus aspect ratio.

Figure 6, Figure 7 and Figure 8 point out that the percolation threshold was greatly affected by the thickness of GNPs, but it had little relation to the barrier height and the conductivity of the polymer, so we calculated the percolation threshold with various aspect ratios of GNPs, as shown in Figure 9. The dotted line represents the theoretical results obtained from the base on the effective medium theory [24] and the exclusion volume [27]. The solid line is from our theoretical calculation. Various symbols are experimental data [18,19,46,48,49,50,51,52,53,54,55]. It can be seen that the result of Monte Carlo calculation was almost the same as that based on the effective medium theory. If the aspect ratio *α* < 0.1, then the curve could be approximately regarded as a straight line with a slope of 1, with a percolation threshold of *Φ_C_* = 1.8*α*. Most pairs of GNPs with less than the cut off distance coincided in space if *l* was far less than *D*, so reducing *l* did not affect the formation of the percolation path. The simulation results showed good agreement with most of the experimental data, while a small part of the experimental data was larger than the theoretical data when *α* was small, which may have been caused by the aggregation of the synthesized GNPs in the polymer. If the aggregation of the inclusion occurred, the original percolation path was broken, which caused the percolation threshold to increase.

## 4. Concluding Remarks

In this paper, a Monte Carlo model was developed to calculate the conductivity of GNP composites. In this model, a GNP is considered to be a point set with a distance from random round faces less than half of the thickness of a GNP. Considering the electron tunneling effect, there was non-zero contact conductance between a pair of GNPs that were close to each other, which played a vital role for the conductivity of the composites. A set of linear equations about the potential of GNPs and CSs were established, where the potential of the CS was calculated by the WoS method. Then, the potential of each GNP was calculated. The conductivity of composites can be obtained by calculating the current through the plane *z* = *d*. There are only four adjustable parameters in the model: polymer conductivity *σ*_0_, barrier height Δ*E*, thickness *l,* and the diameter *D* of GNPs. The conductivity of composites depends on the conductivity of a polymer *σ*_0_ or barrier height Δ*E,* respectively, below or above the percolation threshold, and the percolation threshold is basically determined by the aspect ratio. The relationship between the conductivity and the volume fraction calculated by our theory agrees with the experimental data for four groups under corresponding parameters. The percolation threshold versus aspect ratio curve calculated by our theory was almost consistent with the analytical expression basis of the effective medium theory.
